# Outcomes of surgery for gastric cancer with distant metastases: a retrospective study from the SEER database

**DOI:** 10.18632/oncotarget.14027

**Published:** 2016-12-19

**Authors:** Jiaqi Chen, Yiyao Kong, Shanshan Weng, Caixia Dong, Lizhen Zhu, Ziru Yang, Jing Zhong, Ying Yuan

**Affiliations:** ^1^ Department of Medical Oncology, The Second Affiliated Hospital of Zhejiang University School of Medicine, Hangzhou, Zhejiang Province, China; ^2^ Cancer Institute (Key Laboratory of Cancer Prevention and Intervention, Chinese National Ministry of Education; Key Laboratory of Molecular Biology in Medical Sciences, Zhejiang Province, China), The Second Affiliated Hospital of Zhejiang University School of Medicine, Hangzhou, Zhejiang Province, China; ^3^ Department of Gastroenterology, The Second Affiliated Hospital, School of Medicine and Institute of Gastroenterology, Zhejiang University, Hangzhou, Zhejiang, China

**Keywords:** gastric cancer with distant metastases, surgery, outcomes

## Abstract

**Background:**

The role of surgical therapy in gastric cancer patients with distant metastases remains controversial. This retrospective analysis was performed to identify whether gastric cancer patients with distant metastases might benefit from surgery.

**Patients and methods:**

A total of 5185 patients from the SEER database who were initially diagnosed with histologically confirmed gastric cancer with distant metastases from 2004 to 2009 were included. Patients were divided into the following three groups: patients who underwent resection of both the primary tumor and distant metastatic tumors (‘PMTR’ group), patients who only underwent resection of the primary tumor (‘PTR’ group) and patients who did not undergo any surgery (‘No surgery’ group). We employed the Kaplan-Meier analysis, the log-rank test and multivariate Cox proportional hazards regression models to estimate the survival time of the different groups.

**Results:**

A total of 5185 patients had a median survival time (MST) of 9.0 months. The improvement in survival of the ‘PMTR’ and ‘PTR’ groups was significantly different compared with that of the ‘No surgery’ group (MST, 12.0 *vs* 12.0 *vs* 9.0 months, respectively, *P*<0.001; 1-year survival rate, 49.6% *vs* 49.1% *vs* 30.1%, respectively, *P*<0.001; 3-year survival rate, 12.5% *vs* 15.1% *vs* 5.8%, respectively, *P*<0.001), whereas no significant difference was found between the ‘PMTR’ group and ‘PTR’ group (*P*=0.642). Multivariate Cox proportional analysis showed that surgery was an independent prognostic factor (‘PMTR’, hazard ratio (HR) =0.648, 95% confidence interval (CI) 0.574-0.733, *P*<0.001; ‘PTR’, HR=0.631, 95% CI 0.583-0.684, *P*<0.001).

**Conclusions:**

This retrospective analysis demonstrated that combined PTR and metastasectomy or PTR alone were independent prognostic factors for survival improvement in gastric cancer patients with distant metastases. Because no statistically significant difference in survival was observed between the ‘PMTR’ group and ‘PTR’ group, PTR, which is a more minor surgery, might be more appropriate than PMTR in clinical practice for gastric cancer patients with distant metastases.

## INTRODUCTION

Gastric cancer is the fourth most common type of cancer worldwide and is the second cause of cancer-related death globally [1]. In 2014, in the United States, the numbers of new cases and deaths from gastric adenocarcinoma were estimated to be 22,220 and 10,990, respectively [2, 3]. The incidence of gastric cancer is the second highest in China and is > 20 per 100,000 in men [4]. Because of early tumor detection, curative surgical resection and appropriate adjuvant therapy, the survival of patients with early primary gastric cancer has improved. In a previous study in Japan, the resection rate in patients with early primary gastric cancers was 95.4% and the 5-year survival rate of patients who underwent resection was 70.7% [5]. However, because of the atypical early symptoms, limited popularity of routine gastroscopy examination and other factors, 35% of patients present with evidence of distant metastases at the time of diagnosis [6].As a result, the treatment of gastric cancer patients with distant metastases remains poor. Although systemic chemotherapy with or without new molecular targeting agents is currently the standard treatment modality, the median survival time (MST) of gastric cancer patients with distant metastases is < 12 months and the 5-year survival is < 10% without surgical treatment [7, 8].

Researchers have begun to seek and explore new and more effective treatment options for gastric cancer patients with distant metastases. With advancements in current research and the emergence of a large amount of evidence-based medical findings, some scholars have proposed active surgical treatment for gastric cancer patients with distant metastases [9, 10, 11]. However, because the outcomes of palliative resections in gastric cancer patients with distant metastases are extremely poor [12, 13], the benefits of surgery in these cases remain debatable. Thus, this retrospective analysis was performed to analyze the significance of surgery for gastric cancer with distant metastases and to investigate the prognostic factors associated with surgery to identify candidates with gastric cancer with distant metastases who are most likely to benefit from curative surgical treatment.

## PATIENTS AND METHODS

### Patient selection

The Surveillance, Epidemiology, and End Results Program (SEER) database was sponsored by the National Cancer Institute. In the SEER database, information on cancer cases from 18 population-based cancer registries, which represent approximately 27.8% of the population in the United States, was collected. The database of the SEER program includes information on patient demographics, primary tumor site, tumor histology, stage at initial diagnosis, surgery, radiotherapy, and survival.

Our retrospective study contained 5185 patients from the SEER database (SEER*Stat 8.2.1) who were initially diagnosed with histologically confirmed gastric cancer with distant metastases between 2004 and 2009. The histological type was restricted to adenocarcinoma. Exclusion criteria included patients less than 18 years of age, survival time less than 3 months after confirmed diagnosis, patients with previously diagnosed malignancies, metastasectomy without primary tumor resection or if resection of the primary tumor was unknown, and occult gastric cancer (no evidence of primary tumor). The remaining patients were divided into three groups as follows: patients who underwent resection of both the primary tumor and distant metastatic tumors (‘PMTR’ group), patients who underwent resection of the primary tumor alone (‘PTR’ group) and patients who did not undergo surgery (‘No surgery’ group).

### Statistical analysis

Survival curves, the median survival time (MST), 1-year survival rate and 3-year survival rate were estimated with the Kaplan-Meier method, and the log-rank test was performed to evaluate survival in the different groups. Hazard ratios (HRs) along with 95% confidence intervals (CI) were calculated using the multivariate Cox proportional hazard regression model to determine the influences of other factors including surgery, age, race, gender, tumor site, grade, histological type, T-stage, N-stage, and radiation status, on survival. Statistical tests were two-sided and *P* < 0.05 was considered statistically significant. SPSS 12.0 (SPSS Chicago, IL, USA) software was used for the statistical analysis.

## RESULTS

### Patient characteristics

A total of 5185 eligible patients were included: 322 (6.2%) patients underwent resection of both primary and distant metastatic tumors (‘PMTR’ group), 885 (17.1%) patients underwent PTR alone (‘PTR’ group), and 3978 (76.7%) patients did not undergo any surgery (‘No surgery’ group). A total of 946 (18.24%) patients were over 75 years old and 1843 (35.54%) were male. Patient demographics and characteristics are summarized in Table 1.

**Table 1:** The Characteristics of patients with gastric cancer with distant metastases

Variance	N,%	PMTR,%	PTR,%	No surgery,%	Total
**Total**	5185(100)	322 (6.2)	885 (17.1)	3978(76.7)	
**Age**					
≤75years old	4239(81.76)	268(6.3)	718(16.9)	3253(76.7)	
>75years old	946(18.24)	54(5.7)	167(17.7)	725(76.6)	
*P* value					0.704
**Race**					
white	3778(72.86)	231(6.1)	585(15.5)	2962(78.4)	
black	656(12.65)	35(5.3)	128(19.5)	493(75.2)	
other	739(14.25)	55(7.4)	169(22.9)	515(69.7)	
unknown	12(0.23)	1(8.3)	3(25.0)	8(66.7)	
*P* value					<0.001
**Gender**					
male	1843(35.54)	155(8.4)	320(17.4)	1368(74.2)	
female	3342(64.46)	167(5.0)	565(16.9)	2610(78.1)	
*P* value					<0.001
**Site of tumor**					
body	1809(34.90)	168(9.3)	472(26.1)	1169(64.6)	
cardia	2163(41.70)	92(4.3)	245(11.3)	1826(84.4)	
fundus	218(4.20)	10(4.6)	29(13.3)	179(82.1)	
pylorus	114(2.20)	11(9.6)	38(33.3)	65(57.0)	
stomach	881(17.00)	41(4.7)	101(11.5)	739(83.9)	
*P* value					<0.001
**Grade**					
well	80(1.54)	2(2.5)	11(13.8)	67(83.8)	
moderate	928(17.90)	54(5.8)	154(16.6)	720(77.6)	
poor	3034(58.51)	239(7.9)	625(20.6)	2170(71.5)	
undifferentiated	109(2.10)	13(11.9)	35(32.1)	61(56.0)	
unknown	1034(19.94)	14(1.4)	60(5.8)	960(92.8)	
*P* value					<0.001
**Histological type**					
non-signet-ring cell	3957(76.32)	232(5.9)	610(15.4)	3115(78.7)	
signet-ring cell	1228(23.68)	90(7.3)	275(22.4)	863(70.3)	
*P* value					<0.001
T-stage*					
T_1_	782(15.1)	10(1.3)	39(5.0)	733(93.7)	
T_2_	215(4.1)	10(4.7)	327(16.7)	463(78.6)	
T_3_	676(13.0)	89(13.2)	274(43.2)	279(43.6)	
T_4_	1768(34.1)	198(11.2)	223(27.5)	796(60.7)	
T_x_	1744(33.6)	15(0.9)	22(1.3)	1707(97.9)	
*P* value					<0.001
N-stage*					
N_0_	1449(27.95)	50(3.5)	137(9.5)	1262(84.2)	
N_1_	1611(31.07)	62(3.8)	172(10.7)	1377(85.5)	
N_2_	278(5.36)	64(23.0)	156(56.1)	58(20.9)	
N_3_	540(10.41)	139(25.7)	385(71.3)	16(3.0)	
N_x_	1307(25.21)	7(0.5)	35(2.7)	1265(96.8)	
*P* value					<0.001
**Radiation**					
done	1074(20.71)	67(6.2)	180(16.8)	827(77.0)	
no radiation	4034(77.80)	246(6.1)	692(17.2)	3096(76.8)	
unknown	77(1.49)	9(11.7)	13(16.9)	55(71.4)	
*P* value					0.383

PMTR: patients who underwent resection of both primary tumor and distant metastatic tumors; PTR: patients received primary tumor resection alone; No surgery: patients did not undergo any surgery.

* T-stage and N-stage according to the 7th edition of AJCC TNM staging.

### Survival analyses

**Figure 1:**
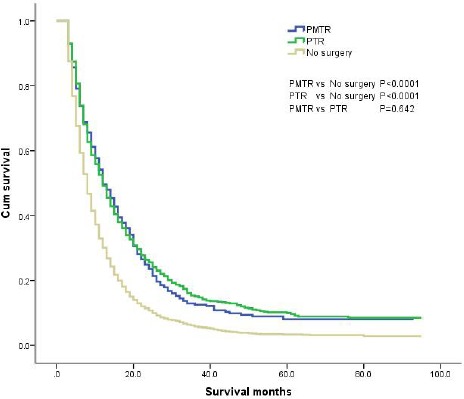
The survival curves of gastric cancer with distant metastases in different grups PMTR: patients who underwent resection of both primary tumor and distant metastatic tumors; PTR: patients received primary tumor resection alone; No surgery: patients did not undergo any surgery.

The results of the Kaplan-Meier analysis and log-rank test showed that a total of 5185 patients had a MST of 9.0 months, a 1-year survival rate of 34.6%, and a 3-year survival rate of 7.9%. Among them, the MST of the ‘PMTR’ group and the ‘PTR’ group was significantly longer compared with that of the No surgery group (12.0 *vs* 12.0 *vs* 9.0 months, *P* < 0.001), whereas no significant difference was observed between the ‘PMTR’ and the ‘PTR’ groups (*P* = 0.642). The 1-year survival rates were 49.6%, 49.1% and 30.1% for the ‘PMTR’, ‘PTR’ and ‘No surgery’ groups, respectively (*P* < 0.001) and the 3-year survival rates were 12.5%, 15.1% and 5.8%, respectively (*P* < 0.001). The survival curves are shown in Figure 1.

### Outcomes of the different subgroups

**Figure 2:**
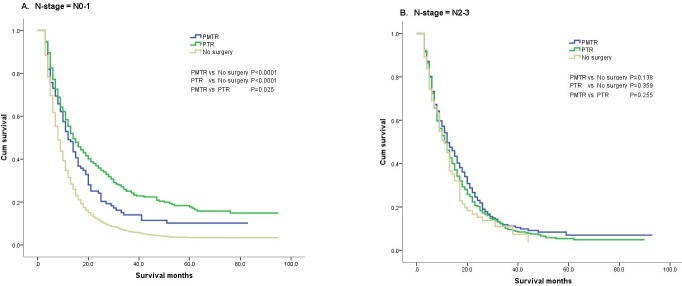
The survival curves of three groups in N-stage subgroups A. N_0-1_ subgroup; B: N_2-3_ subgroup.

We compared the survival benefit of patients according to the subgroups, which accounted for age, race, gender, grade, tumor site, histological type, T-stage, N-stage and radiation status, by Kaplan-Meier analysis and log-rank test. Among the different subgroups, the survival benefits of patients in the ‘PMTR’ and ‘PTR’ groups were better than those seen in patients in the ‘No surgery’ group (Table 2). Specifically, in the N_0-1_ subgroup, the results showed that the survival improvement of patients in the ‘PMTR’ and ‘PTR’ groups was significantly higher compared with that of patients in the ‘No surgery’ group (MST, 12.0 *vs* 14.0 *vs* 8.0 months, *P* < 0.001; 1-year survival rate, 49.1% *vs* 55.4% *vs* 31.4%, *P* < 0.001; 3-year survival rate, 14.0% *vs* 25.1% *vs* 6.2%, *P* < 0.001). Moreover, patients with stage N_0-1_ cancer in the ‘PTR’ group had an increased survival benefit compared with those in the ‘PMTR’ group (*P* < 0.05). However, among those with stage N_2-3_ cancer, no statistically significant differences were found in the MST, the 1-year or the 3-year survival rate among the three groups (*P* > 0.05) (Figure 2).

**Table 2:** Survival rate(%) and MST(Months) of patients with gastric cancer with distant metastases

	PMTR			PTR			No Surgery			*P*
	Survival rate(%)		MST	Survival rate(%)		MST	Survival rate(%)		MST	
	1Y	3Y		1Y	3Y		1Y	3Y		
**Total**	49.6	12.5	12.0	49.1	15.1	12.0	30.1	5.8	9.0	<0.001
**Age**										
≤75y	52.9	13.6	14.0	51.3	15.9	13.0	30.9	6.2	8.0	<0.001
>75y	32.9	6.4	9.0	39.4	11.5	8.0	26.5	4.3	7.0	<0.001
**Race**										
white	51.9	11.4	14.0	47.2	15.4	12.0	30.2	5.4	8.0	<0.001
black	38	10.4	11.0	54.1	14.2	13.0	26.1	5.2	7.0	<0.001
other	46.2	16.4	12.0	51.6	14.1	13.0	32.5	8.6	8.0	<0.001
**Gender**										
male	55.9	13.8	15.0	51.1	16.5	13.0	30.7	5.6	8.0	<0.001
female	42.9	11	11.0	45.5	12.5	11.0	28.8	6.3	8.0	<0.001
**Site of tumor**										
body	47.8	13.7	12.0	58.2	15.6	12.0	30.1	6.4	80.0	<0.001
cardia	60.6	13.8	16.0	57.3	23.3	13.0	32.1	6.6	9.0	<0.001
fundus	40	0	11.0	58.6	18.7	14.0	26.2	2.8	7.0	0.007
pylorus	43.6	10.9	12.0	41.7	19.3	8.0	24.6	1.5	6.0	0.004
stomach	36.6	9.8	10.0	44.1	19.3	12.0	26.4	4.2	7.0	<0.001
**Grade**										
well	50	0	10.0	45.5	0	11.0	35.5	3.1	9.0	0.899
moderate	64.2	28.5	17.0	66.8	21.7	17.0	35.1	6.7	9.0	<0.001
poor	47.6	9.7	12.0	45.2	12.9	11.0	28.9	5.3	8.0	<0.001
undifferentiated	25.2	0	11.0	48.6	15.7	12.0	35.4	10.3	9.0	0.145
**Histological type**										
non-signet-ring cell	53.7	15.1	14.0	53	16.7	13.0	30.8	6.2	8.0	<0.001
signet-ring cell	38.8	5.1	11.0	40.3	11.3	10.0	27.4	4.4	7.0	<0.001
T-stage*										
T_1-2_	60	25	14.0	63.3	35.6	24.0	30.7	6.1	8.0	<0.001
T_3-4_	49.4	11.8	12.0	47.7	12.8	12.0	32	6	8.0	<0.001
N-stage*										
N_0-1_	49.1	14	12.0	55.4	25.1	14.0	31.4	6.2	8.0	<0.001
N_2-3_	49.6	11.2	12.0	46.2	9.6	11.0	45	11	11.0	0.272
**Radiation**										
done	63.9	24.5	17.0	58.3	20.5	16.0	31.1	5.6	8.0	<0.001
no radiation	46.7	9.5	12.0	46.3	13.4	11.0	29.7	5.8	8.0	<0.001

PMTR: patients who underwent resection of both primary tumor and distant metastatic tumors; PTR: patients received primary tumor resection alone; No surgery: patients did not undergo any surgery.

* T-stage and N-stage according to the 7th edition of AJCC TNM staging.

The superiority in the ‘PMTR’ and ‘PTR’ groups was completely diminished compared with the ‘No surgery’ group only in the subgroups that contained patients with well differentiated and undifferentiated tumors. Considering the far smaller number of patients who underwent surgery in these two subgroups, the results should be interpreted with caution.

### Multivariate analyses for survival

The multivariate Cox proportional hazard regression analysis showed that surgery was an independent prognostic factor (‘PMTR’, hazard ratio (HR) = 0.577, 95% confidence interval (CI) 0.491-0.677, *P* < 0.001; ‘PTR’, HR = 0.559, 95% CI 0.493-0.633, *P* < 0.001). We also analysed all the aforementioned factors in the subgroups and found that age, histological type, N-stage and radiation status were also independent prognostic factors (Table 3).

**Table 3:** Multivariate analysis (Cox Proportional Hazard Model) of overall survival for patients with gastric cancer with distant metastases

Variable (reference)	Wald	HR	95.0% CI for HR	*P*
Surgery (no surgery)				
PMTR	45.222	0.577	0.491-0.677	<0.001
PTR	84.258	0.559	0.493-0.633	<0.001
Age (≤75 years old)	27.819	1.351	1.208-1.511	<0.001
Race (white)				
black	0.149	1.025	0.902-1.165	0.700
other	2.111	0.915	0.812-1.031	0.146
Gender (male)	0.638	1.038	0.948-1.137	0.424
Site of tumor (body)				
cardia	0.459	0.965	0.872-1.069	0.498
fundus	2.248	1.174	0.952-1.449	0.134
pylorus	0.155	1.056	0.807-1.382	0.693
stomach	0.078	1.021	0.882-1.182	0.779
Grade (well)				
moderate	0.869	0.865	0.638-1.173	0.351
poor	0.148	1.060	0.787-1.427	0.701
undifferentiated	0.014	0.978	0.672-1.423	0.906
Histology type (non-signet-ring cell)	13.467	1.214	1.095-1.347	<0.001
T-stage (T_3-4_)*	3.439	0.908	0.820-1.005	0.064
N-stage (N_2-3_)*	6.010	0.858	0.760-0.970	0.014
Radiation (no radiation)	6.989	0.871	0.786-0.965	0.008

PMTR: patients who underwent resection of both primary tumor and distant metastatic tumors; PTR: patients received primary tumor resection alone; No surgery: patients did not undergo any surgery.

* T-stage and N-stage according to the 7th edition of AJCC TNM staging.

### Prognostic factors associated with surgery

The multivariate Cox proportional hazard regression analysis showed that in the ‘PMTR’ group, age and radiation status were independent prognostic factors, while in the ‘PTR’ group, in addition to age and radiation status, T-stage and N-stage were also independent prognostic factors (Table 4).

**Table 4:** Multivariate analysis (Cox Proportional Hazard Model) of overall survival of subgroups in group ‘PMTR’, group ‘PTR’ and group ‘No surgery’ respectively

Variance	PMTR			PTR			No surgery		
	*P*	HR	95.0% CI	P*	HR	95.0% CI	P**	HR	95.0% CI
**Age**(≤75years old)									
>75years old	0.004	1.675	1.182-2.374	0.005	1.336	1.090-1.637	0.001	1.286	1.108-1.492
**Race**(white)									
black	0.927	0.979	0.620-1.546	0.480	0.918	0.724-1.164	0.329	1.085	0.921-1.277
other	0.866	0.970	0.680-1.384	0.152	0.863	0.705-1.056	0.322	0.920	0.780-1.085
Gender									
female	0.166	1.212	0.923-1.591	0.226	1.107	1.939-1.305	0.819	0.986	0.872-1.115
**Site of tumor**(body)									
cardia	0.964	0.993	0.724-1.361	0.652	1.043	0.870-1.249	0.400	0.942	0.820-1.082
fundus	0.240	1.508	0.760-2.991	0.906	0.974	0.626-1.515	0.129	1.224	0.943-1.588
pylorus	0.380	1.374	0.676-2.793	0.819	0.952	0.623-1.454	0.698	1.084	0.722-1.628
stomach	0.796	1.060	0.682-1.648	0.741	0.955	0.725-1.257	0.694	1.039	0.857-1.261
**Grade**(well)									
moderate	0.464	0.466	0.060-3.596	0.033	0.505	0.270-0.948	0.955	1.010	0.707-1.443
poor	0.681	0.681	0.090-5.162	0.182	0.663	0.362-1.213	0.326	1.191	0.841-1.686
undifferentiated	1.244	1.244	0.149-10.379	0.107	0.564	0.281-1.132	0.960	0.987	0.599-1.626
**Histological type** **(non-signet-ring cell)**									
signet-ring cell	0.110	1.278	0.946-1.727	0.364	1.086	1.909-1.299	0.006	1.223	1.059-1.411
T-stage*(T_3-4_)									
T_1-2_	0.282	0.732	0.415-1.292	0.001	0.587	0.425-0.810	0.710	0.979	0.875-1.095
N-stage*(N_2-3_)									
N_0-1_	0.949	0.991	0.740-1.326	0.001	0.751	0.631-0.894	0.179	1.218	0.913-1.624
**Radiation**(no radiation)									
done	0.025	0.682	0.488-0.954	0.004	0.745	0.609-0.911	0.733	0.977	0.856-1.116

PMTR: patients who underwent resection of both primary tumor and distant metastatic tumors; PTR: patients received primary tumor resection alone; No surgery: patients did not undergo any surgery.

HR= hazard ratio, CI = confidence interval.

* T-stage and N-stage according to the 7th edition of AJCC TNM staging.

## DISCUSSION

In recent years, surgical treatment for gastric cancer with distant metastases has remained controversial. Although systemic chemotherapy is the standard treatment strategy, the MST of these patients is only approximately 12 months, and long-term survival beyond 5 years is rare [14, 15]. Recently, it was reported that in selected cases, especially in patients with gastric cancer with liver metastases, aggressive surgical treatment may lead to unexpected results [16, 17, 18, 19, 20]. In a review based on 19 studies, Kerkar et al. reported that the 5-year survival rate of gastric cancer patients with liver metastases who underwent liver resection was 26.5% (range: 0-60%) [16]. Similarly, Kodera et al. analysed 17 studies that involved patients with gastric cancer with liver metastases and showed that the MST ranged from 9.0 to 38.8 months among patients who underwent surgical resection [17]. Recently, two meta-analyses also verified that hepatectomy might be associated with the significant improvement in overall survival [18, 19]. In an analysis of 39 studies that involved patients with gastric cancer with liver metastases who underwent hepatectomy, Markar et al. found that the median 1-year, 3-year, and 5-year survival rates were 68%, 31%, and 27%, respectively [18]. In another meta-analysis of 11 observational studies, Martella et al. reported a significantly higher survival rate in the patients who underwent the most aggressive surgery for liver metastases (HR = 0.54, 95% CI 0.46-0.95) compared with patients who underwent palliative treatment only [19]. Studies of gastric cancer patients with distant metastases other than those to the liver who underwent curative surgery are scarce. In a retrospective study initiated by HAN et al., gastric cancer patients with distant metastases, including metastases to the liver, para-aortic lymph nodes, peritoneum and ovary, were enrolled, and those who were good responders to induction chemotherapy underwent curative R_0_ resection [20]. Finally, the median survival of patients was as high as 22.9 months. In the meta-analysis, a survival benefit and clinical significance were also found for palliative gastrectomy for metastatic gastric cancer (HR = 0.62; 95%CI 0.49-0.78; *P* < 0.0001) [21]. All these studies demonstrated that surgery was a potential approach to improve the outcome of selected patients with metastatic gastric cancer.

Recently, the opposite result was reported in the REGATTA randomized controlled trial [22]. REGATTA was an open-label, randomized, phase 3 trial with 44 participating centres and hospitals in Japan, South Korea, and Singapore. Patients aged 20-75 years old with advanced gastric cancer confined to either the liver (H1), peritoneum (P1), or para-aortic lymph nodes (16a1/b2) and who had a single non-curable factor were enrolled and randomly assigned (1:1) in each country to chemotherapy alone or gastrectomy (D1 lymphadenectomy) followed by chemotherapy. The overall survival at 2 years as the primary endpoint for all randomly assigned patients was 31.7% (95% CI 21.7-42.2) for those assigned to chemotherapy alone compared with 25.1% (16.2-34.9) for those assigned to gastrectomy plus chemotherapy. The median overall survival was 16.6 months (95% CI 13.7-19.8) for patients assigned to chemotherapy alone and 14.3 months (11.8-16.3) for those assigned to gastrectomy plus chemotherapy (hazard ratio 1.09, 95% CI 0.78-1.52; one-sided *P* = 0.70). Based on the result of this trial, compared with chemotherapy alone, resection of the primary tumor plus chemotherapy did not improve survival. In contrast to the aforementioned retrospective study, the majority of the enrolled patients in this trial experienced peritoneal metastasis (75%). This undesirable composition of the enrolled patients may have led to the negative result in this trial.

However, not all patients with metastatic gastric cancer obtained a survival benefit from surgery, and these candidates for surgery shared some general characteristics. In the study by Samarasam et al., 107 of 151 patients underwent surgical resection and 44 underwent non-resectional surgery [23]. The MST of the patients who underwent surgical resection was 24.0 months, but the MST was 12.0 months for those who underwent non-resectional surgery. The patients were divided into four groups according to widespread tumor growth (T+), unresectable lymph node involvement (L+), liver metastasis (H+) and peritoneal metastasis (P+). All patients with one positive sign underwent resection, and the resultant MST was 24.3 months. Patients with two positive signs had a survival advantage in favour of surgical resection, in contrast to those who underwent non-resectional surgery (13.0 *vs* 8.0 months). When three signs (6.0 *vs* 12.0 months) and four signs (2.0 *vs* 2.6 months) were present, the survival advantage of patients who underwent surgical resection disappeared. Similarly, Hartgrink et al. found that among 156 patients who underwent palliative resection and 77 who did not undergo resection, the MST was greater in the resection group (8.1 *vs* 5.4 months; *P* < 0.001) [24]. A significant difference in survival benefit was found in patients with one metastatic site between the resection group and the non-resection group (MST, 10.5 *vs* 6.7 months; *P* = 0.034) while no significant survival advantage was observed in patients who underwent resection of two or more metastatic sites (5.7 *vs* 4.6 months, *P* = 0.084). In addition, they found that although patients aged over 70 years gained a 3-month improvement in survival after surgery, the morbidity and perioperative mortality rates in this older age group were much higher (50% and 20%, respectively).

The results of the current study were similar to those described above. Compared with patients who did not undergo surgery, those who underwent PMTR or PTR experienced significant improvements in survival. Furthermore, in the subgroup analyses, with the exception of the N_2_-N_3_ stage subgroup, the factors of age, race, gender, tumor site, grade, histological type, T-stage, and radiation status did not influence the surgery-associated benefits seen in these patients. This may imply that patients with N_2_-N_3_ stage cancer would not benefit from surgery. However, no significant difference was found in terms of a survival benefit between the patients who underwent PMTR and those who underwent PTR. Additionally, in clinical practice, PMTR may lead to more treatment complications and risks. This suggests that, in the same cases, PTR may represent a better option compared with PMTR.

Thus far, many investigators have agreed that age, histological type, N-stage and radiation status were independent prognostic factors [16, 17, 18, 19, 20, 21]. In our study, using a multivariate analysis, we arrived at the same conclusions and found that surgery was an additional independent prognostic factor. This implied that compared with those who did not undergo surgery, patients who underwent surgery might obtain a survival benefit and experience a significant decrease in the risk of death. Similarly, younger patients, those with non-signet-ring cell adenocarcinoma, those with early N-stage cancers and those who received radiation therapy might be more likely to experience a survival benefit compared with other patients. In addition, younger patients and those who received radiation were more likely to benefit from PMTR. However, in the ‘PTR’ group, younger patients, those with early T-stage tumors, those with early N-stage tumors, and those who received radiation therapy were more prone to undergo PTR.

Some limitations may have influenced the results of our study. First, our study had limitations that are inherent to the methodology of retrospective analyses, including selection bias and potential confounders. Thus, we integrated the data of the following patient groups to reduce bias as much as possible because of insufficient sample capacity: T_1_ and T_2_ were integrated into the T_1-2_ subgroup, T_3_ and T_4_ were integrated into the T_3-4_ subgroup, N_0_ and N_1_ were integrated into the N_0-1_ subgroup, and N_2_ and N_3_ were integrated into the N_2-3_ subgroup. Second, information such as the performance status of the patients, the site and number of metastases, whether patients underwent synchronous or metachronous surgery, chemotherapy status, and comorbidities were not included in the SEER database. Finally, the determination of the T-stage and N-stage of patients who underwent surgery depended on the postoperative pathologic results, while for those who did not undergo surgery, they were determined according to the imaging results.

In conclusion, we sought to evaluate whether gastric cancer patients with distant metastases would benefit from surgery. The results showed that surgical treatment was able to improve effective survival time except patients with N_2_-N_3_ stage cancer. From the results of this study, we considered that patients who were younger and those with early stage primary tumors might obtain a greater survival benefit from surgical treatment than others. Additionally, radiation therapy may strengthen the survival benefit that is gained from surgical treatment. Furthermore, the improvements in the survival of patients who underwent PMTR and PTR were not statistically significant. As a result of more treatment complications and risks that are associated with PMTR in clinical practice, PTR may represent a better option for gastric cancer patients with distant metastases. Our study was a retrospective analysis with limitations and our conclusions should be further validated by a more prospective randomized trial.
